# International collaborative study for the calibration of a proposed World Health Organization International Standard for thromboplastin, human, recombinant: a report from the International Federation of Clinical Chemistry and Laboratory Medicine (IFCC)—Scientific and Standardization Committee (SSC)/ISTH working group on prothrombin time/international normalized ratio standardization

**DOI:** 10.1016/j.rpth.2026.106793

**Published:** 2026-06-12

**Authors:** Michelle P. van der Helm, Claudia J.J. van Rijn, Shanti S. Baktawar, Charmane F. Abdoel, Steve Kitchen, Michelle Bryant, Paula Brown, Armando Tripodi, Erica Scalambrino, Marigrazia Clerici, Petra Herbel, Anja Jünschke, Piet Meijer, Anne Stavelin, Craig Thelwell, Christa M. Cobbaert, Antonius M.H.P. van den Besselaar

**Affiliations:** 1 Coagulation Reference Laboratory (CRL), Department of Clinical Chemistry and Laboratory Medicine, Leiden University Medical Center (LUMC), Leiden, the Netherlands; 2 Sheffield Haemophilia and Thrombosis Centre, Royal Hallamshire Hospital, Sheffield, United Kingdom; 3 Fondazione Istituto di Ricovero e Cura a Carattere Scientifico (IRCCS) Ca’ Granda Maggiore Hospital Foundation, Angelo Bianchi Bonomi Hemophilia and Thrombosis Centre, Milano, Italy; 4 Roche Diagnostics GmbH, Mannheim, Germany; 5 External quality Control of diagnostic Assays and Tests (ECAT) Foundation, Voorschoten, Netherlands; 6 The Norwegian Organisation for Quality Improvement of Laboratory Examinations, Bergen, Norway; 7 Therapeutic Reference Materials, Medicines and Healthcare products Regulatory Agency, National Institute for Biological Standards and Control, Potters Bar, United Kingdom

**Keywords:** international normalized ratio, international standard, prothrombin time, thromboplastin

## Abstract

**Background:**

The availability of International Reference Preparations for thromboplastins, later on referred to as International Standards (ISs) for thromboplastins is essential for calibration of prothrombin time (PT) measurement systems and for the international normalized ratio (INR). It is proposed to replace the current fifth IS for thromboplastin, human, recombinant (coded rTF/16) and the fifth IS for thromboplastin, rabbit, plain (coded RBT/16) by a single IS for defining a reference measurement system for PT/INR tests, in agreement with the concept of metrological traceability.

**Objectives:**

This study aimed to determine the international sensitivity index (ISI) for the proposed sixth IS for thromboplastin, human, recombinant (coded 24/114).

**Methods:**

An international collaborative study was carried out for the assignment of the ISI to the proposed IS by calibration against the current fifth IS for thromboplastin, human, recombinant. PT measurements were performed by 8 trained operators in 4 laboratories (2 operators in each laboratory) using the harmonized manual tilt tube technique. The World Health Organization calibration procedure was followed.

**Results:**

ISI values from 8 operators varied from 1.04 to 1.12, and the mean ISI for the proposed IS coded 24/114 was 1.08 (between-operator coefficient of variation, 2.8%).

**Conclusion:**

The material coded 24/114 has been accepted by the World Health Organization as the sixth IS for thromboplastin, human, recombinant. Extensive prior training and external quality assessment surveys for the operators reduced the between-operator coefficient of variation. In this era of metrological traceability, a sustainable reference measurement system has been developed for PT/INR based on the harmonized manual tilt tube technique and a new IS.

## Essentials

• The PT is measured for control of therapy with vitamin K antagonists.
• The PT is transformed to the INR for global exchange.
• An IS has been prepared for metrological traceability of the INR.
• The proposed IS has been accepted by the WHO Expert Committee on Biological Standardization.

## 1 Introduction

Treatment with vitamin K antagonists (VKA) must be monitored to adjust the dose if necessary. The primary laboratory method for monitoring of VKA is the prothrombin time (PT) test. The PT test is performed with a thromboplastin reagent and a method or instrument to determine the coagulation time in seconds. Various types of thromboplastin are prepared commercially, and in order to be able to interpret the results of the PT test, it is essential that each reagent is correctly calibrated [1]. This ensures that the results of tests with different products and batches can be compared. A procedure for the calibration of thromboplastins using a logarithmic plot of PTs has been developed. With this procedure, the definition of a calibration parameter called international sensitivity index (ISI) became feasible. It became possible to express PT results on a common scale, ie, the international normalized ratio (INR) [1] with the following formula:

INR = (PT_patient_/MNPT)^ISI^.

where MNPT (mean normal prothrombin time) is the geometric mean of the PTs of the healthy adult population. For practical purposes, the geometric mean of the PT calculated from at least 20 fresh samples from healthy individuals, including both male and female, is a reliable approximation of MNPT [1].

Two International Standards (ISs) for thromboplastin are currently available for calibration of secondary standards or commercial thromboplastin preparations: the fifth IS for thromboplastin, rabbit, plain (coded RBT/16) and the fifth IS for thromboplastin, human, recombinant (coded rTF/16) [2]. In general, the calibration of a given thromboplastin reagent is more precise (ie, with a lower coefficient of variation [CV] of the calibration line slope) if performed against an IS of similar composition and from the same species. In the past, plain rabbit thromboplastins were calibrated against RBT/16 and plain human thromboplastins against the human recombinant material rTF/16. Thromboplastins of bovine or rabbit brain combined with adsorbed bovine plasma (Owren type) were also calibrated against the prevailing IS for thromboplastin rabbit, plain [1]. However, recently it has been recognized that the calibration procedure for replacement of coexisting ISs may result in biased ISI values [3]. In addition, it was recognized that a sustainable metrologically traceable calibration hierarchy for INR should be based on an international protocol for value assignment with a single primary reference thromboplastin, in agreement with the principles of metrological traceability (ISO 17511:2020) [4,5].

Stocks of both rTF/16 and RBT/16 are now running low and replacement is required. A candidate replacement material has been prepared for thromboplastin, human, recombinant (International Reference Preparation [IRP] coded 24/114). The calibration of the candidate reference thromboplastins should be carried out as part of international multicenter collaborative studies using fresh coumarin patient citrate plasmas and fresh normal healthy individual citrate plasmas and using manual techniques for PT determination. In the past, the ISI assigned to each replacement material was the mean of the ISIs obtained by calibration with both existing ISs [1]. With each successive replacement of an IS for thromboplastin, the uncertainty of the ISI increased [3]. To avoid further drifting of the ISI and to reduce the measurement uncertainty of the INR, unequivocal and sustainable metrologically traceable calibration hierarchies were defined for the INR measured in citrate plasma and whole blood [4].

Originally, World Health Organization (WHO) international reference thromboplastins were referred to as IRP for thromboplastins. Since 1996, the term IRP for thromboplastin was changed by WHO to IS for thromboplastin. According to ISO 17511, the term standard is defined as the “realization of the definition of a given quantity, with stated quantity value and associated measurement uncertainty, used as a reference” [5]. In spite of the discordance between the WHO term IS for thromboplastin and the ISO 17511 definition of the term standard, we will use the WHO term IS.

In the present study, PT measurements were performed with the harmonized manual tilt tube (MTT) technique [6]. There are several reasons for using the MTT rather than an automated instrument for ISI assignment with a primary IS. First, it should be appreciated that the ISI value depends on the procedure for PT measurement [7]. Replacing the MTT by an automated instrument will result in a change of the ISI value. Different instruments will generate different ISI values. Second, the proposed primary IS is expected to last for at least 20 years and should be used for calibration of secondary reference procedures only. The Reference Measurement Procedure used with the primary IS should last equally long. In general, commercial automated instruments have a shorter life time than ∼20 years. Third, automated PT instruments consume much more reagent than the MTT because of the so-called dead volume in the instrument, which is not economical, considering the high cost of the primary IS. Finally, in the PT/INR calibration hierarchy, only a limited number of dedicated laboratories will use the IS for calibration of secondary reference procedures and manufacturer’s selected measurement procedures [4]. Previous and the present studies have demonstrated that training of MTT operators in dedicated PT/INR calibration laboratories is effective [8]. There is no need for end-user clinical laboratories to apply the MTT or Reference Measurement Procedure.

The present report deals with the results of an international collaborative multicenter calibration study organized by a working group under the auspices of the International Federation of Clinical Chemistry and Laboratory Medicine (IFCC) and Scientific and Standardization Committee (SSC) of the International Society on Thrombosis and Haemostasis (ISTH) for standardization of the PT/INR.

## 2 Materials and Methods

### 2.1 Thromboplastin reagents

The fifth IS for thromboplastin, human, recombinant (coded rTF/16) was obtained from the Medicines and Healthcare products Regulatory Agency (MHRA), National Institute for Biological Standards and Control, Potters Bar, United Kingdom. The proposed replacement IS was prepared from purified recombinant human tissue factor produced in *Escherichia coli*, combined with synthetic phospholipids, calcium, buffers, and stabilizers. The bulk material was shipped by the manufacturer (Siemens) to MHRA for further processing. Aliquots (1.0 mL) of the material were freeze dried in glass ampoules as agreed previously for all ISs [9]. After sealing, samples were selected for determination of fill mass, dry weight, residual moisture, and oxygen head space. Each ampoule is to be reconstituted with 2.0 mL of purified water. The freeze-dried material received the code 24/114. Production details for the freeze-dried material obtained by the MHRA are shown in Table 1.

**Table 1:** Production specifications for the proposed WHO sixth International Standard provided by the Medicines and Healthcare products Regulatory Agency (MHRA).

Descriptive items	WHO sixth International Standard thromboplastin human recombinant, coded: 24/114 technical preparations	
CAS and CARN number	NA	
Trivial name: main component(s) and/or property/properties	Proposed WHO sixth International Standard for Thromboplastin, Human, Recombinant (24/114)	
Biological name for labeling with component name	Recombinant human tissue factor produced in *Escherichia coli*, combined with synthetic phospholipids, calcium, buffers, and stabilizers	
Production details	Manufacturing site	MHRA, Potters Bar, United Kingdom
	Date of fill	May 3, 2024
	No. of containers available	23,278
	Custodian and storage site	MHRA, Potters Bar, United Kingdom
Acceptable performance commensurate with its use	Mean fill mass (CV)	1.0075 g (0.1271%), *n* = 771
	Mean dry weight (CV)	0.14789 g (0.23%), *n* = 6
	Mean residual moisture (CV)	0.20% (150%), *n* = 12
	Mean oxygen head space (CV)	0.31% (42.24%), *n* = 12
Checking procedure along with tolerance intervals, eg, for absence of interfering components	NA	
Utensils and special cleaning procedures	NA	
Storage conditions	Storage temperature	−20 °C
Shelf life	“Reference materials are held at the MHRA South Mimms laboratories within assured, temperature-controlled storage facilities and they should be stored on receipt as indicated on the label. This standard has been produced according to WHO specifications and as per WHO policy an expiry date has not been assigned. Accelerated degradation studies have indicated that this material is suitably stable, when stored at −20 °C or below, for the assigned values to remain valid until the material is withdrawn or replaced.”	
Disposal	According to local procedures for biological waste products	
Hazard class	“The product is not classified as hazardous according to Regulation (EC) 1272/2008 as amended.”	

CARN, CAS Registry Number; CAS, Chemical Abstracts Service; CV, coefficient of variation; NA, not applicable; WHO, World Health Organization.

### 2.2 Lyophilized control plasmas

Four lyophilized control plasmas were obtained from Technoclone GmbH. One lyophilized plasma (coded A) was obtained from healthy donors. Three plasmas (coded B, C, and D) were prepared from donors who had been on long-term anticoagulation therapy with VKAs, covering the range of ∼1.5 to 4.5 INR as described previously [10].

### 2.3 Design of the collaborative study for ISI value assignment

Four European laboratories (Leiden, Mannheim, Milan, and Sheffield), being active members of the abovementioned IFCC-ISTH Working Group on PT/INR standardization, were invited to participate in the collaborative study. Eight operators in the selected laboratories (2 operators in each laboratory) participated in wet workshops with the aim to harmonize all details of the MTT technique, and the operators further participated in external quality assessment trials set up for the MTT technique [6,8]. The candidate material (24/114) and the current IS (rTF/16) were tested in each laboratory by each operator with the refined harmonized MTT, according to a study protocol with detailed instructions (Supplementary Figure 1A and B). The study was approved by each laboratory’s medical ethics committee, and informed consent was obtained from each subject. Test plasmas were freshly prepared from healthy (normal) subjects and patients on long-term therapy with VKA, stably anticoagulated for 6 weeks. The 2 operators in each laboratory analyzed the same series of plasma samples independently. According to the current WHO guidelines, patients’ samples with INRs in the range 1.5 to 4.5 should be used for calibration [1]. The majority of patients are treated with a therapeutic target range of 2.0 to 3.0. It is therefore difficult to recruit patients with INRs in the upper part of the 1.5 to 4.5 range. Tripodi et al. [11] and Kitchen et al. [12] showed that using samples with INR of <4.0 had only a marginal effect on the results of ISI calibration. In the present study, participants were instructed to select patient plasmas with PTs corresponding to an INR interval from 1.5 to 4.0. PT measurements were performed in duplicate by each operator. In addition to the fresh plasmas, 4 lyophilized control plasmas were included. To account for the effect of interday variation, PT measurements were performed in each laboratory on 10 different days. Participants were instructed to include on each day plasmas from 2 healthy individuals and 6 patients treated with VKA. Healthy individuals and patients had to be different on each day. To minimize the effect of preanalytical plasma instability on the relationship between thromboplastins, the order of testing was changed each day. The order of testing was specified in the data collection form. The collaborative study was carried out from October 2024 until November 2024.

### 2.4 Statistical methods

#### 2.4.1 Calculation of ISI for candidate materials

The statistical methods have been described in the guidelines for thromboplastins and plasma used to control oral anticoagulant therapy with VKAs [1]. The sequence of steps in the statistical evaluation for each participant’s calibration is as follows:

1. Calculate preliminary orthogonal regression line (20 normal samples + 60 patient samples).
2. Detect outliers defined as points with a perpendicular distance (D) >3 residual SDs (RSDs) from the preliminary line.
3. Remove outliers in 1 step and recalculate the orthogonal regression line (normal samples + patient samples) and ISI of the candidate as the slope of the regression line multiplied with the ISI of the IS rTF/16.
4. Calculate each patient’s INR using the PT determined with the IS.
5. Calculate each patient’s INR using the PT determined with the candidate material and ISI from step 3.
6. Calculate each patient’s mean INR from steps 4 and 5.
7. Remove patients with mean INR of <1.50 or >4.00.
8. Recalculate the orthogonal regression line for the remaining normal samples + patient samples represented by the equation *Y = a* + *bX* in which *Y* is the natural logarithm of PT determined with the IS rTF/16 and *X* is the natural logarithm of PT determined with the candidate IS. The ISI of the candidate is obtained as the slope (ie, *b*) of the regression line multiplied with the ISI of the IS rTF/16.

In the abovementioned sequence of steps, it is necessary to calculate intermediate quantities, eg, MNPT and regression residuals. It is necessary to round the quantities to a defined number of decimal places. The decision to reject measurements as outliers or as INR of <1.50 or >4.00 may depend on the rounding of intermediate quantities. The numbers of decimal places are given in Supplementary Table 1.

#### 2.4.2 Mean ISI and between-laboratory CV

The arithmetic mean and between-laboratory SD of ISI assessments was calculated. The between-laboratory CV was calculated as the SD divided by the mean ISI and multiplied by 100. The SEM was calculated as SD/√*n* in which *n* is the number of ISI assessments. A *t*-distribution was used to calculate the 95% CI for the mean ISI ± *t* × SD/√*n* [13].

#### 2.4.3 Validity of ISI

Each and every ISI assessment was checked for validity by using 2 criteria. First, the within-operator CV (CV_b_) of the slope of the orthogonal regression line for normal samples + patient samples should be ≤3% [1]. ISI assessments with a within-laboratory CV_b_ of the slope >3% should be rejected. Second, the adequacy of the ISI model was checked for linearity. While there is good evidence that the calibration relationship defined in a double-logarithmic plot of PTs is usually linear and that the same line represents data points for both patients and healthy subjects, the possibility of departure from these assumptions cannot be ruled out. To assess the magnitude of INR deviations, the orthogonal regression line was calculated for patients’ samples only as follows:

*Y* = *a'* + *b'X*.

where *Y* is the natural logarithm of patient’s PT determined with the IS rTF/16, and *X* is the natural logarithm of patient’s PT determined with the proposed IS. It is assumed that the line *Y* = *a'* + *b'X* represents the “true” relationship between measurements performed with each of the 2 thromboplastins. INRs calculated with the line *Y* = *a'* + *b'X*, were compared with INRs calculated with the ISI obtained from patients and normal samples. The average difference between the 2 INRs is a measure of the INR bias. In case of marked INR deviation, the assignment of an ISI would not be meaningful. For practical purposes, the assignment of an ISI is acceptable if INRs calculated with the ISI do not differ by >10%, in the INR range 2 to 4.5 [1].

#### 2.4.4 Repeatability of PT/INR

Repeatability of PT/INR was determined from the duplicate measurements by each operator. Each PT measurement was transformed to INR using each operator’s specific MNPT and the mean ISI for rTF/16 (ie, 1.11) and candidate 24/114 (ie, 1.08) respectively. The repeatability CV (CV_R_) was calculated as follows:

$${\text{CV}}_{R}=100\times \sqrt{(}\sum {\left(d/m\right)}^{2}\times {\left(2n\right)}^{-1})$$

where *d* is the difference between 2 duplicate measurements, *m* is the mean INR of the 2 measurements, and *n* is the number of differences [14].

#### 2.4.5 Variability of INR. The variation of INR between rTF/16 and candidate 24/114 was calculated as follows:

$${\text{CV}}_{T}=100\times \sqrt{(}\sum {\left(d/m\right)}^{2}\times {\left(2n\right)}^{-1})$$

where *d* is the difference between a measurement with rTF/16 and a measurement with candidate 24/114, *m* is the mean INR of the 2 measurements, and *n* is the number of differences.

#### 2.4.6 Between-operator variation of lyophilized plasma INR

The mean PT of each of the lyophilized plasmas was calculated for each operator. INR was calculated from the mean PT, the MNPT of the operator, and the mean ISI of all operators. The between-operator CV was calculated from the SD and mean INR of all operators. Between-operator CVs obtained in the present study were compared with those obtained in a previous multicenter study [2]. The Mann–Whitney U-test was used to compare the 2 groups of CVs.

#### 2.4.7 Commutability assessment

For assessment of commutability of the lyophilized control plasmas, the method of regression analysis and the 95% prediction interval was used [15]. According to this method, a 95% prediction interval is calculated based on the results for fresh native clinical samples. Then, the position of reference materials (ie, the lyophilized control plasmas) relative to the 95% prediction interval is assessed. If the mean of the reference material results falls outside the 95% prediction interval for the native patient samples, the reference material is considered noncommutable.

## 3 Results

### 3.1 ISI value assignment

Each operator completed a full 10-day series of PT measurements independently. The number of outliers (ie, points with distance D from the regression line > 3 RSD) and samples with INR of <1.50 or >4.00 were calculated for each operator (Table 2). The orthogonal regression lines and ISI values and MNPT calculated after exclusion of outliers (D > 3 RSD) and samples with INR of <1.50 or >4.00 are shown in Table 3. The mean ISI was 1.08 with a between-operator SD of 0.03 (CV, 2.8%). The SEM ISI obtained by 8 operators were 0.011. The 95% CI of the mean ISI was 1.05 to 1.11. The within-operator CV of the regression line slope (CV_b_) was <3% for each operator (Table 3).

**Table 2:** Number of outliers with a perpendicular distance from the regression line D > ±3 RSD and number of VKA-treated patient samples with mean INR of <1.50 or >4.00.

Laboratory/operator	Original total No. of samples	Fifth International Standard rTF/16 and proposed Sixth International Standard 24/114		
		D > ±3 RSD	INR < 1.50	INR > 4.00
Laboratory 1: operator 1	80	1	2	1
Laboratory 1: operator 2	80	1	2	1
Laboratory 2: operator 3	80	2	—	2
Laboratory 2: operator 4	80	1	—	3
Laboratory 3: operator 5	80	—	1	—
Laboratory 3: operator 6	80	—	—	—
Laboratory 4: operator 7	80	1	1	3
Laboratory 4: operator 8	80	1	1	2

INR, international normalized ratio; RSD, residual SD; VKA, vitamin K antagonist.

**Table 3:** Orthogonal regression lines *Y* = *a* + *bX* and ISI values for the candidate WHO Sixth International Standard coded 24/114.

Laboratory/operator	*N*	Intercept (*a*)	Slope (*b*)	CV_b_ (%)	ISI	MNPT (s)
Laboratory 1: operator 1	76	0.0697	0.9952	1.2	1.10	11.41
Laboratory 1: operator 2	76	0.0452	0.9964	1.4	1.11	11.72
Laboratory 2: operator 3	76	0.1470	0.9743	1.1	1.08	11.17
Laboratory 2: operator 4	76	0.1447	0.9718	1.2	1.08	11.04
Laboratory 3: operator 5	79	0.2326	0.9359	1.7	1.04	11.52
Laboratory 3: operator 6	80	0.2138	0.9374	1.5	1.04	11.36
Laboratory 4: operator 7	75	0.0847	1.0106	1.1	1.12	10.89
Laboratory 4: operator 8	76	0.1949	0.9694	1.1	1.08	10.67
Average					1.08	11.22
CV (%)					2.8	3.1

*N* is the number of normal and patients’ samples after removal of outliers.

CV_b_, within-operator coefficient of variation of the orthogonal regression line slope; CV, between-operator coefficient of variation; ISI, international sensitivity index; MNPT, mean normal prothrombin time; WHO, World Health Organization.

The deviations of the INR calculated with the ISI for the true INR of 2.00, 3.00, and 4.00 are shown in Table 4. All operators showed deviations less than 10%. In conclusion, the validity of the ISI for the proposed IS was confirmed.

**Table 4:** Orthogonal regression lines *Y* = *a'***+***b'X* for the candidate WHO Sixth International Standard coded 24/114.

Laboratory/operator	*N*	Intercept (*a'*)	Slope (*b'*)	INR deviation (%)		
				INR = 2.00	INR = 3.00	INR = 4.00
Laboratory 1: operator 1	56	0.0100	1.0129	0.9	0.1	−0.4
Laboratory 1: operator 2	56	0.0049	1.0085	0.5	0.0	−0.3
Laboratory 2: operator 3	56	−0.1472	1.0607	4.0	0.6	−1.7
Laboratory 2: operator 4	56	−0.1859	1.0692	4.6	0.8	−1.8
Laboratory 3: operator 5	59	0.2871	0.9199	−0.8	−0.1	0.4
Laboratory 3: operator 6	60	0.1481	0.9564	1.1	0.3	−0.3
Laboratory 4: operator 7	55	0.1269	0.9980	−0.2	0.7	1.2
Laboratory 4: operator 8	56	0.1587	0.9802	0.6	0.2	−0.2

*N* is the number of patients’ samples after removal of outliers. Mean deviation (%) of INR calculated with ISI by comparison with INR calculated with orthogonal regression line *Y = a'* + *b'X*.

INR, international normalized ratio; ISI, international sensitivity index; WHO, World Health Organization.

### 3.2 Repeatability of INR

The repeatability of the INR from duplicate measurements on the fresh patient samples is shown in Table 5.

**Table 5:** Repeatability of INR (CV_R_) and INR between-thromboplastin variation (CV_T_) determined with fresh VKA-treated patient samples after removal of outliers.

Operator	No. of samples	Mean INR with rTF/16	CV_R_ (%) with rTF/16	Mean INR with candidate 24/114	CV_R_ (%) with candidate 24/114	CV_T_ (%)
1	56	2.71	1.1	2.66	1.3	4.0
2	56	2.64	1.8	2.58	1.7	4.3
3	56	2.86	2.0	2.88	1.5	3.5
4	56	2.83	1.2	2.86	1.3	4.0
5	59	2.70	3.4	2.81	2.0	5.7
6	60	2.78	1.6	2.91	1.6	5.5
7	55	2.88	1.8	2.76	1.5	4.3
8	56	2.83	1.5	2.85	1.4	3.2

CV, coefficient of variation; INR, international normalized ratio; VKA, vitamin K antagonist.

### 3.3 Variation of INR between measurement procedures

The variation of INR between the 2 measurement procedures—rTF/16 and candidate 24/114—is shown in Table 5.

### 3.4 Lyophilized control plasmas

For each of the lyophilized control plasmas and for each operator, the INR was calculated with each operator’s local MNPT and the mean ISI for rTF/16 (ie, 1.11) and the mean ISI for the proposed IS 24/114 (ie, 1.08). Table 6 shows the mean INR and the between-operator CV. Mean INR values for control plasmas B, C, and D obtained with proposed IS 24/114 were lower than those obtained with rTF/16, suggesting that these plasmas may not be commutable. The ISI calculated with the 4 lyophilized control plasmas (ISI_L_) was higher than the ISI calculated with the fresh plasma samples (compare Table 6 with Table 3). WHO guidelines recommend validation of lyophilized plasma to be used for calibration of PT/INR measurement systems [1]. By using the recommended validation procedure, we found that the average INR bias between rTF/16 (ISI = 1.11) and candidate 24/114 (ISI_L_ = 1.24) ranged from 11% to 20%, which is beyond the maximum value of 10% recommended by the WHO guidelines [1]. The lack of commutability of the lyophilized control plasmas is also illustrated by a plot of the results obtained by operator number 1 (Figure) in which the points representing the results for the lyophilized control plasmas are located beyond the 95% prediction interval for the results for the fresh plasma samples obtained by the same operator. Similar results were obtained by the other operators. Despite potential lack of commutability of the lyophilized plasmas and despite the use of different lots of lyophilized plasmas in different studies, the INR between-operator CVs for the lyophilized plasmas in the present study are significantly lower (*P* < .05, Mann–Whitney U-test) than those obtained in a previous multicenter calibration study [2]. This is also reflected in a median CV% change from 6.4% to 3.2%, suggesting that the overall operator performance has been improved in the present study.

**Table 6:** Mean INR and between-operator CV for lyophilized control plasma A, B, C, and D determined with Fifth International Standard rTF/16 and proposed International Standard 24/114.

Operator	INR determined with rTF/16				INR determined with candidate IS 24/114				ISI_L_	CV_b_
	A	B	C	D	A	B	C	D		
1	1.00	2.26	3.43	5.52	0.93	1.82	2.78	4.03	1.25	3.0
2	1.00	2.20	3.30	5.32	0.96	1.76	2.70	3.86	1.28	4.3
3	0.98	2.19	3.34	5.45	0.98	1.91	2.91	4.24	1.25	2.8
4	1.03	2.22	3.41	5.51	0.98	1.92	2.96	4.33	1.21	2.5
5	0.98	2.11	3.20	5.16	0.95	1.81	2.78	4.05	1.23	3.1
6	0.99	2.10	3.19	5.12	0.92	1.80	2.75	4.02	1.19	2.5
7	1.00	2.25	3.49	5.61	1.01	1.88	2.85	4.13	1.31	2.6
8	1.03	2.21	3.36	5.45	1.00	1.88	2.88	4.19	1.24	3.1
Average	1.00	2.19	3.34	5.39	0.97	1.85	2.82	4.11	1.24	
CV (%)	2.1	2.7	3.2	3.3	2.7	3.2	3.2	3.6	3.0	

International sensitivity index (ISI_L_) for the proposed International Standard was calculated with the mean prothrombin times determined with the 4 lyophilized control plasma samples. CV_b_ is the within-operator CV of the slope of the orthogonal regression line determined with the mean prothrombin times of the 4 lyophilized control samples.

CV, coefficient of variation; INR, international normalized ratio.

**Figure:**
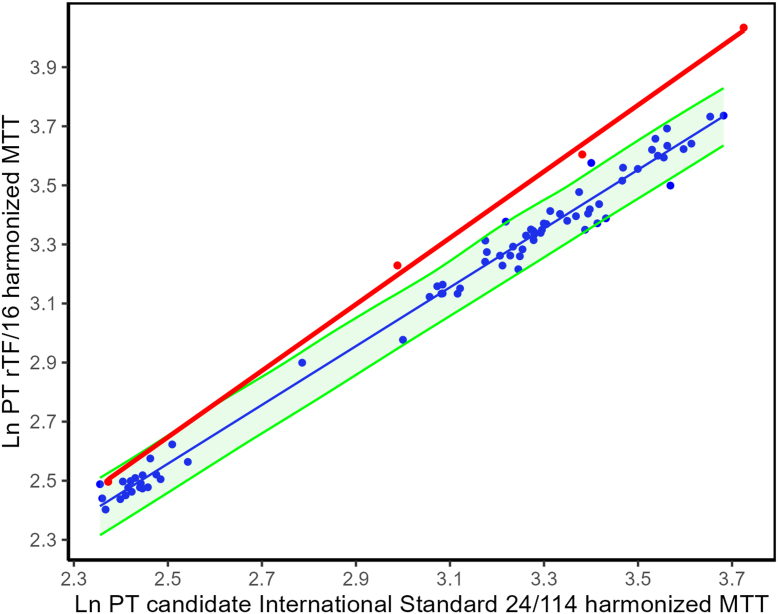
Scatterplot of log-transformed prothrombin times (PTs) obtained by operator number 1. On the vertical axis, the results with International Standard rTF/16 are given. On the horizontal axis, the results with the proposed International Standard 24/114 are given. The blue symbols represent the results obtained with fresh plasma samples. The blue line represents the orthogonal regression line for the fresh samples. The green area represents the 95% prediction interval for the fresh samples. The red symbols represent the results (average of results obtained on 10 days) obtained with the 4 lyophilized control plasmas. The red line represents the orthogonal regression line for the lyophilized plasmas. This figure was created with R statistical software, version 4.4.1 (2024-06-14 Universal C Run Time). MTT, manual tilt tube.

## 4 Discussion

The purpose of the present study was to assign an ISI value to a proposed new IS for thromboplastin, recombinant, human. ISI assignment was performed by calibration against the current fifth IS for thromboplastin, recombinant, human, plain, coded rTF/16. In contrast to the previously recommended procedure in which calibration was performed with all existing WHO ISs [16], only 1 IS (ie, rTF/16) was used in the present study. The current IS for thromboplastin, rabbit, plain (coded RBT/16) was not used and should not be replaced by a new IS of the same species. An important reason for using only 1 IS (ie, rTF/16) in the present study is that, in previous studies, an ISI bias was observed between human and rabbit ISs [2,3,17]. The ISI obtained by calibration with the IS, recombinant, human, plain was lower than the ISI obtained by calibration with the IS, rabbit, plain, and the difference between the ISI values increased with each replacement [3]. The origin of the ISI bias observed in previous international collaborative calibration studies should be investigated separately. The ISI bias is probably not due to deterioration of the thromboplastins. It has been shown that the ISI values for the ISs RBT/05 and rTF/09 appeared to be stable for 38 months at storage temperatures of −20, −70, and −150 °C [18]. Long-term stability of the proposed new IS will be monitored in the coming years. Accelerated degradation studies were performed on the new IS showing acceptable results (Supplementary Tables 2 and 3). Acceptable stability after reconstitution of the new IS was achieved between 60 and 180 minutes after reconstitution (Supplementary Figure 2). Homogeneity of the new IS was acceptable (Supplementary Tables 4-7).

Another important reason for proposing a single IS for thromboplastin is that the metrologically highest placed measurement procedure in the calibration hierarchy shall be identified and shall define the highest level of metrological traceability for the measurement system [5]. A sustainable metrologically traceable calibration hierarchy for INR should be based on an international protocol for value assignment with a single primary reference thromboplastin and the harmonized MTT technique for clotting time determination [4].

The proposed replacement of the current ISs for human and rabbit thromboplastins by a single IS for human thromboplastin will result in a minor change of the ISI (and hence INR) for commercial human and rabbit thromboplastin PT systems. The magnitude of this change is presently being assessed and will be reported in the near future. An example of an INR change as a result of the previous replacement of IS rTF/09 by rTF/16 has been published by Kitchen et al. [19]. These investigators observed a 4.3% difference in INRs determined with Recombiplastin 2G in 2 surveys performed before and after replacement of the ISs, in agreement with the predicted change.

The between-operator CV of the ISI for the proposed IS coded 24/114 was 2.8% (Table 3) and was low in comparison with the between-operator CV in previous multicenter calibration studies [2,17]. For example, the between-operator CV of the ISI assignment for 2 previous candidate ISs for thromboplastin, human, plain, ranged from 3.5% to 6.5% [17]. It seems that the between-operator CV is improved in the present study when compared with previous ISI calibrations. The improvement may be explained by the prior training of the operators in workshops and by participation of the operators in an external quality assurance program for the harmonized MTT technique [6,8]. Preliminary training of the operators did not take place in previous collaborative calibration studies. The between-operator CV of the ISI (Table 3) can be used to estimate the standard measurement uncertainty of the INR determined by any 1 of trained MTT operators, as described recently [20].

The ISI assessment was considered adequate for calculation of the INR (Table 4). The greatest INR deviation (ie, 4.6%) was observed by operator number 4 at an INR level of 2.00. According to the WHO guidelines [1], a deviation up to 10% is acceptable. We do not know whether the INR deviation observed by operator number 4 is due to preanalytical or analytical factors. The present study was the first to assess INR repeatability (CV_R_) by operators using fresh patients’ samples (Table 5). There were differences between operators. Operator number 1 showed the lowest values for CV_R_ (1.1%-1.3%), but operator number 5 displayed the highest values (2.0%-3.4%). The origin of the differences is not known.

In the present study, we evaluated the between-thromboplastin variation (CV_T_) of the fresh patients’ samples for each operator (Table 5). CV_T_ ranged from 3.2% to 5.7%. Part of CV_T_ may be explained by CV_R_ as the operator with the higher CV_T_ also had the higher CV_R_. CV_T_ is also influenced by the difference in composition of the 2 thromboplastins being compared. Although rTF/16 and the proposed IS are both prepared from recombinant human tissue factor, these reagents have multiple different components such as phospholipid, calcium, buffer, and stabilizers (Table 1). In general, a greater difference in composition is associated with a greater INR variability because there is interaction between reagent composition and the patient’s sample composition [4].

The 4 lyophilized control plasmas included in the present study were used to assess the between-operator variation of the INR (Table 6). The CVs with the proposed IS ranged from 3.3% to 3.6%, where the CV increased with increasing INR. The CVs in the present study are, on average, much lower than those observed in the previous study where CV values ranged from 3.2% to 8.1%. The lower CVs in the present study may be explained by the harmonization of the MTT technique and the preliminary training of the operators.

The mean INR values for the 4 lyophilized plasmas calculated with the mean ISI were significantly lower with the proposed IS when compared with the corresponding values obtained with the current IS rTF/16 (Table 6). If the 4 lyophilized plasmas were used for calculation of the ISI of the proposed IS, the average ISI_L_ would be ∼15% higher than the mean ISI obtained with fresh plasma samples (compare Table 6 with Table 3). A bias of 15% is unacceptable for ISI calibration because it results in a mean INR bias greater than the maximum value of 10% recommended by the WHO guidelines [1]. Although we did not perform a formal assessment of the commutability of the 4 lyophilized plasmas with other reagents [21], we suggest that manufacturers of commercial thromboplastins and lyophilized standard plasmas should perform commutability studies when INR values are to be assigned to these plasmas. The manufacturer or supplier of certified standard plasmas should clearly specify the set of reagent/instrument combinations for which their materials may be reliably used [22,23]. Further work is needed to assess the usefulness and limitations of lyophilized plasmas for ISI calibration.

This work was submitted for approval by the SSC Subcommittee on Thrombosis and Antithrombotic Therapies. On approval, the work was submitted to the WHO Expert Committee on Biological Standardization (ECBS). The candidate material coded 24/114 was established as the sixth WHO IS for thromboplastin, human, recombinant, by the ECBS in October 2025.

## 5 Conclusion

The material coded 24/114 has been accepted as the sixth WHO IS for thromboplastin, human, recombinant, with an assigned ISI value of 1.08. Prior training and external quality assessment surveys for the operators reduced the between-operator CV of the ISI. In this era of metrological traceability, a sustainable reference measurement system has been developed for PT/INR based on the harmonized MTT and a new IS for thromboplastin.
