# Supplementary material for: International collaborative study for the calibration of a proposed World Health Organization International Standard for thromboplastin, human, recombinant: a report from the International Federation of Clinical Chemistry and Laboratory Medicine (IFCC)—Scientific and Standardization Committee (SSC)/ISTH working group on prothrombin time/international normalized ratio standardization
Source: Res Pract Thromb Haemost. 2026 Jun 12;10(4):106793. doi: 10.1016/j.rpth.2026.106793 (PMC13380524; doi:10.1016/j.rpth.2026.106793)

**Supplementary Material**

**Refined harmonized MTT procedure**

Measurement instructions according to the actual updated* harmonized MTT procedure [1]

*Protocol harmonized MTT; updated after workshop November 9th 2021

- Take care that the actual room temperature is 20 -22 °C (RT).
- Fill the water level in the water bath, i.e. 1 cm underneath the edge of the bath (harmonization of distance of tube’s horizontal position to water bath during tilting, i.e. 2 – 10 cm).
- Keep empty test tubes in a vertical position in a rack in the water bath at 37°C (± 0.1 ˚C) for at least 4 minutes at a depth of 3.5 cm before reagent and plasma samples are transferred successively with micropipettes.
- Mark the pipette at the level corresponding to the upper edge of the test tube to determine the exact level of pipetting the plasma, i.e. 1 cm above the thromboplastin reagent in the tube
- Harmonize the speed of pipetting the reagent and plasma into the glass tube (avoid splashing by letting the tip of pipette rest against the wall of the tube); the stop watch must be started when the operator begins to push the button of the pipette; the time of the complete push movement being 0.5 s
- Pipette 200 μL of thromboplastin into glass test tube at 37°C and incubate for 2 minutes.
- Pipette 100 μL of not prewarmed citrate plasma **1 cm above the level of thromboplastin** with the tip resting against the wall of the tube and start the stopwatch with the other hand immediately.
- After pipetting the plasma into the glass tube shake gently with the tube immersed in the water to mix the contents. Put the tube in the rack in the water bath and lay down the pipette.
- Take the tube out of the rack, the test tube shall be kept manually in the water covering 5 cm of the tube before starting the tilting (Figure S1A)
- Let your hand rest on the edge of the water bath (harmonization of distance of tube’s horizontal position to water bath during tilting, i.e. 2 – 10 cm).

Figure S1A Figure S1B

- Start manual tilting of the tube 7 seconds after adding the plasma to the thromboplastin and starting the stopwatch
- Tilt the tube through an angle of nearly 90° by taking the tube out of the water for 2 seconds and putting it back in the water for 1 second (Figure S1B). The tube should not be stationary during this cycle but continuously tilted with the operator's hand resting on the edge of the water bath.
- Then the cycle shall be repeated until the clot is formed. In the horizontal position, the tube is kept not more than 10 cm and not <2 cm above the water level (Figure S1B).
- Before the mixture clots, the operator observes the mixture flowing from the bottom to three-quarters of the length of the tube in the nearly horizontal position and back to the bottom
- When clotting commences, the speed of flowing is reduced. At this point, the operator is keen to observe the final stopping of the flow.
- When flow is stopped, the operator stops the timer and records the clotting time in seconds in two decimal places.

**Calculation of ISI and rounding of intermediate quantities**

Table S1. (Derived) quantities to be calculated for the calibration of a PT measurement system against a PT reference measurement system and the required number of decimal places.

| **Quantity** | **Derived quantity** | **Number of decimal places** |
| --- | --- | --- |
| Manual Prothrombin time (s) |  | 2 |
|  | Slope of preliminary orthogonal regression line (all PT data) | 4 |
|  | Standard deviation of regression residuals | 4 |
|  | 3 × Standard deviation of regression residuals | 3 |
|  | Regression residuals | 3 |
|  | MNPT (s) | 2 |
|  | Preliminary ISI of system to be calibrated (after removal of outliers) | 3 |
|  | INR calculated with preliminary ISI (after removal of outliers) | 2 |
|  | INR calculated with International Reference Measurement Procedure (RMP) | 2 |
|  | Mean INR | 2 |
|  | Slope of orthogonal regression line (after removal of mean INR<1.50 and >4.00) | 4 |
|  | Final ISI of system to be calibrated | 2 |
|  | Standard deviation of slope of final regression line | 4 |
|  | Coefficient of variation of slope of final regression line (%) | 1 |

**Stability of the proposed International Standard**

**Accelerated degradation study**

Tissue factor is a glycoprotein that needs the association with phospholipids for full expression of its procoagulant activity. Lyophilized tissue factor preparations (i.e. thromboplastin) for PT determination usually contain many other components such as residual water which may influence the stability of the reagent. In general, the long-term stability of lyophilized tissue factor from human or animal brain stored at low temperature is excellent [9]. The stability of lyophilized biological materials may be predicted from accelerated degradation tests. The purpose of an accelerated degradation test is to measure the relative rates of potency loss at several temperatures and to extrapolate the rate to the desired temperature of storage. For the deterioration of lyophilized tissue factor preparations complex degradation kinetics are expected because tissue factor is a lipoprotein [10]. It may be difficult to predict the stability of tissue factor at low temperature from the results of an accelerated degradation test. Nevertheless, the results of an accelerated degradation test may be useful to assess the relative stability under transportation conditions at various temperatures. The accelerated degradation test is a standard procedure to check the stability of lyophilized thromboplastin preparations.

For the accelerated degradation study, nine ampoules of the proposed International Standard 24/114 were stored at -20 °C, +4 °C, +20 °C, +37 °C, and +45 °C. After storage of the ampoules at these temperatures for 1, 2, and 3 months, they were reconstituted and tested with two deep-frozen pooled plasmas (one normal, coded NP, and one coumarin plasma obtained from patients treated with VKA , coded AP). The relative short duration of the accelerated degradation study was due to the short time between preparation of the proposed International Standard and the deadline for testing of the ampoules. The tests were performed with the harmonized MTT by one operator. For each storage temperature and storage time, 3 ampoules were used. Each ampoule was tested in a single PT determination. For each storage temperature and time, a clotting time ratio (PT ratio) was calculated from the PT of the coumarin plasma AP divided by the mean PT of the normal plasma NP. As the PT ratio is used to calculate the INR of the plasma AP, a potential change of the PT ratio reflects a potential change of the ISI. Linear regression lines were calculated for the PT and PT ratio as a function of the storage time. Pearson correlation coefficients were used to test the change of PT or PT-ratio with storage time. A significance level of 5% was used.

Linear regression lines and Pearson correlation coefficients for the PT are shown in Table 6. The Pearson correlation coefficients were not significant for the results obtained with ampoules stored at -20 °C, +4 °C, +20 °C, and +45 °C. Only for the results obtained with the ampoules stored at +37 °C the Pearson correlation coefficient was significant.

Linear regression lines and Pearson correlation coefficients for the PT ratio are shown in Table 7. The Pearson correlation coefficients were not significant for the results obtained with ampoules stored at -20 °C, +4 °C, and +37 °C. Only for the results obtained with the ampoules stored at +20 °C and +45 °C the Pearson correlation coefficients were significant. In conclusion, storage of ampoules at low temperatures, i.e. -20 °C and +4 °C, warrants stability for at least 3 months. Storage at higher temperatures, i.e. , +20 °C, +37 °C and +45 °C may result in functional deterioration of the proposed International Standard. Therefore, for shipment of the proposed International Standard at ambient temperature certain precautions may be advised. The shipment duration should be limited to a few days. Cooling packs should be included in the parcel to avoid adverse effects of elevated temperature.

Since the proposed International Standard will be used for many years, it is important to monitor the stability in real time. A protocol for stability monitoring of the proposed International Standard is now in preparation and will be started in the next months. The protocol is based on the protocol used for previous International Standards [11].

**Stability after reconstitution**

To assess the stability of the proposed International Standard after reconstitution, ampoules were reconstituted with purified water and kept at room temperature (+22 ± 1°C) for various time intervals and tested in a PT test. The time intervals were 25, 50, 75, 100, 125, 150, 175, 200, 225 and 250 minutes, respectively. Two deep frozen pooled plasmas (one normal, coded NP, and one coumarin plasma obtained from patients treated with VKA, coded AP) were used for the PT tests. The tests were performed in duplicate with the harmonized MTT. Pearson correlation coefficients were used to test the change of PT with incubation time. A significance level of 5% was used. The results are shown in Figure 2. The change of the PT of coumarin plasma AP with time was significant when all measurements between 25 and 250 minutes were included (Figure 1A). If only PT measurements between 60 and 180 min were included, the change was not significant (Figure 1B). The change of the PT of normal plasma NP with time was not significant when all measurements between 25 and 250 minutes were included (Figure 1C), and when measurements between 60 and 180 minutes were included (Figure 1D). In conclusion, acceptable stability of the proposed International Standard 24/114 is achieved between 60 and 180 minutes after reconstitution.

**Homogeneity after reconstitution**

In addition to assessment of the homogeneity of the fill of the ampoules by weight (Table 1), functional homogeneity of the fill was examined after reconstitution of randomly selected ampoules. For the functional homogeneity test 12 ampoules of the proposed International Standard were reconstituted and used with the harmonised MTT on two pooled plasmas (one normal, coded NP, and one coumarin plasma obtained from patients treated with VKA, coded AP). The tests were performed in duplicate. Analysis of variance (ANOVA) was done with PT results obtained with each plasma. ANOVA was not significant (p > 0.05) with the normal plasma NP (Table 8) and with the coumarin plasma AP (Table 9). In addition, a homogeneity check according to ISO 13528 [12] was performed on the PT results. The between-samples CV with plasma NP was 0.9% and with plasma AP was 0% (see Tables 10 and 11, respectively). The different CVs can be explained by the difference in within-sample standard deviation which was higher for plasma AP in comparison to plasma NP. In conclusion, the functional homogeneity of the proposed International Standard was acceptable.

TABLE S2. Accelerated degradation of proposed International Standard 24/114. Linear regression lines of PT (PT=A+B.t) and Pearson correlation of PT with storage time (t in months). Each line and correlation coefficient was calculated with 9 measurements. A significant correlation at the 5% significance level is indicated by an asterisk. NP = Normal Plasma. AP = Abnormal Plasma

| **Plasma** | **Temperature (°C)** | **A (seconds) ± standard error** | **B (s/month) ± standard error** | **Pearson correlation coefficient** | **Pearson Significance** |
| --- | --- | --- | --- | --- | --- |
| NP | - 20 | 11.86 ± 0.28 | -0.280 ± 0.131 | -0.623 | 0.073 |
| NP | + 4 | 11.39 ± 0.25 | -0.098 ± 0.110 | -0.307 | 0.421 |
| NP | + 20 | 11.70 ± 0.31 | -0.337 ± 0.144 | -0.661 | 0.052 |
| NP | + 37 | 11.18 ± 0.11 | -0.235 ± 0.058 | -0.876 | 0.010* |
| NP | + 45 | 10.43 ± 0.23 | 0.083 ± 0.105 | 0.286 | 0.455 |
| AP | - 20 | 27.14 ± 0.57 | -0.430 ± 0.27 | -0.523 | 0.148 |
| AP | + 4 | 26.33 ± 0.39 | -0.100 ± 0.181 | -0.238 | 0.599 |
| AP | + 20 | 25.82 ± 0.42 | 0.015 ± 0.195 | 0.029 | 0.941 |
| AP | + 37 | 27.05 ± 0.56 | -0.828 ± 0.261 | -0.768 | 0.016* |
| AP | + 45 | 24.56 ± 0.78 | -0.672 ± 0.362 | -0.574 | 0.106 |

TABLE S3. Accelerated degradation of proposed International Standard 24/114. Linear regression lines of PT-ratio (PT-ratio = A_R_ + B_R_.t) and Pearson correlation of PT-ratio with storage time (t in months). Each line and correlation coefficient was calculated with 8 measurements. A significant correlation at the 5% significance level is indicated by an asterisk

| **PT-ratio** | **Temperature (°C)** | **A_R_ ± standard error** | **B_R_ (month^-1^) ± standard error** | **Pearson corr. coefficient** | **Pearson Significance** |
| --- | --- | --- | --- | --- | --- |
| PT(AP): PT(NP) | - 20 | 2.29 ± 0.051 | -0.019 ± 0.024 | -0.285 | 0.457 |
| PT(AP): PT(NP) | + 4 | 2.31 ± 0.035 | 0.012 ± 0.016 | 0.267 | 0.488 |
| PT(AP): PT(NP) | + 20 | 2.20 ± 0.038 | 0.073 ± 0.018 | 0.841 | 0.004* |
| PT(AP): PT(NP) | + 37 | 2.44 ± 0.051 | -0.040 ± 0.023 | -0.543 | 0.131 |
| PT(AP): PT(NP) | + 45 | 2.35 ± 0.058 | -0.080 ± 0.027 | -0.751 | 0.020* |

TABLE S4. Functional homogeneity of proposed International Standard. ANOVA results for PT determinations with normal plasma (NP). Sig. = Significance. df = degrees of freedom. F = mean square (between groups)/mean square (within groups).

|  | **Sum of Squares** | **df** | **Mean Square** | **F** | **Sig.** |
| --- | --- | --- | --- | --- | --- |
| **Between Groups** | 0.543 | 11 | 0.049 | 1.718 | 0.183 |
| **Within Groups** | 0.345 | 12 | 0.029 |  |  |
| **Total** | 0.888 | 23 |  |  |  |

TABLE S5. Functional homogeneity of proposed International Standard. ANOVA results for PT determinations with coumarin plasma (AP). Sig. = Significance. df = degrees of freedom. F = mean square (between groups)/mean square (within groups).

|  | **Sum of Squares** | **df** | **Mean Square** | **F** | **Sig.** |
| --- | --- | --- | --- | --- | --- |
| **Between Groups** | 6.340 | 11 | 0.576 | 0.899 | 0.567 |
| **Within Groups** | 7.697 | 12 | 0.641 |  |  |
| **Total** | 14.037 | 23 |  |  |  |

**TABLE S6.** Homogeneity check on proposed International Standard with plasma NP according to ISO 13528 (Annex B).

| Number of samples | 12 |
| --- | --- |
| General average PT (s) | 11.29 |
| SD of sample averages (s) | 0.157 |
| Within-samples SD (s) | 0.170 |
| Between-samples SD (s) | 0.102 |
| Between-samples CV (%) | 0.9 |

**TABLE S7.** Homogeneity check on proposed International Standard with plasma AP according to ISO 13528 (Annex B).

| Number of samples | 12 |
| --- | --- |
| General average PT (s) | 29.70 |
| SD of sample averages (s) | 0.537 |
| Within-samples SD (s) | 0.801 |
| Between-samples SD (s) | 0.000 |
| Between-samples CV (%) | 0.0 |

FIGURE S2. Stability after reconstitution of proposed International Standard 24/114. (A) Abnormal Plasma AP (all points 25-250 min), p<0.01. (B) Abnormal Plasma AP (stable interval between red lines: 60-180 min), p =0.95. (C) Normal Plasma NP (all points 25-250 min), p=0.18. (D) Normal Plasma NP (stable interval between red lines: 60-180 min), p=0.82. Linear regression lines are shown in each panel.


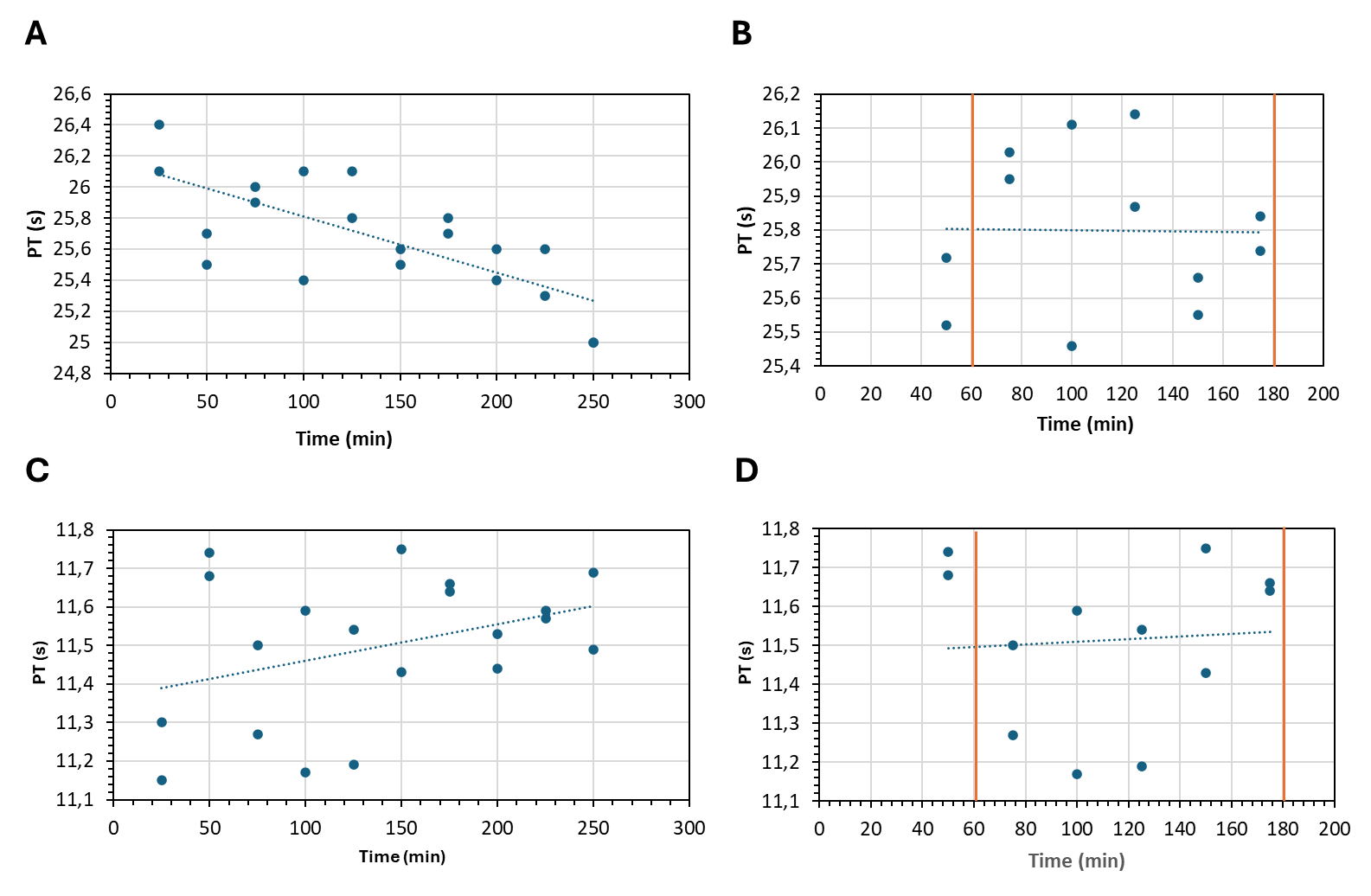

Supplement: Supplemental Material [file mmc1.docx]
